# Cross-sectional associations between psychological traits, and HPV vaccine uptake and intentions in young adults from the United States

**DOI:** 10.1371/journal.pone.0193363

**Published:** 2018-02-23

**Authors:** Aaron M. Scherer, Heather Schacht Reisinger, Marin L. Schweizer, Natoshia M. Askelson, Angela Fagerlin, Charles F. Lynch

**Affiliations:** 1 Department of Internal Medicine, Division of General Internal Medicine, University of Iowa, Iowa City, Iowa, United States of America; 2 Center for Comprehensive Access and Delivery Research and Evaluation, Iowa City Veterans Affairs Health Care System, Iowa City, Iowa, United States of America; 3 Department of Community and Behavioral Health, University of Iowa, Iowa City, IA, United States of America; 4 Department of Population Health Sciences, University of Utah, Salt Lake City, Utah, United States of America; 5 Center for Informatics Decision Enhancement and Surveillance, Salt Lake City Veterans Affairs Health Care System, Salt Lake City, Utah, United States of America; 6 Department of Epidemiology, University of Iowa, Iowa City, Iowa, United States of America; University of Rhode Island, UNITED STATES

## Abstract

Human papillomavirus (HPV) is the most prevalent sexually transmitted infection worldwide and can lead to the development of genital warts, and cancers throughout the body. Despite the availability of HPV vaccines for over a decade, uptake in the United States among adolescents and young adults remains well below national targets. While most efforts to improve HPV vaccine uptake have rightly focused on adolescents, there is still a tremendous opportunity to improve vaccination among young adults who have not been vaccinated against HPV. To that end, we report an exploratory examination of associations between HPV vaccination status and intentions with psychological traits that may impact HPV vaccine uptake with a national, demographically diverse sample of young adults (N = 1358) who completed an online survey. These psychological traits conceptually mapped onto motivations to: 1) understand health-related information, 2) deliberate, 3) manage uncertainty, and 4) manage threats. We found notable gender differences for the association of these motivations and vaccination status. For women, higher interest in and ability to understand health-related information seemed to distinguish those who reported receiving the HPV vaccine from those who did not. For men, less need to deliberate and greater needs to manage threat and uncertainty seemed to be the distinguishing motives for those who reported receiving the HPV vaccine compared to those who did not. Results for vaccination intentions were less consistent, but there was some evidence to indicate that, regardless of gender, greater health-related information interest and understanding and need to manage uncertainty and threats were associated with increased intention to receive the HPV vaccine, while greater need to deliberate was associated with decreased vaccination intentions. These results suggest that there are psychological differences that are associated with HPV vaccination decisions and that these motivations should be considered in efforts to improve HPV vaccine uptake.

## Introduction

Human papillomavirus (HPV) is the most prevalent sexually transmitted infection worldwide and can lead to the development of genital warts and cancers throughout the body, including the cervix, penis, head and neck, and rectum [1,2]. Despite the availability of HPV vaccines for over a decade, uptake in the United States remains well below the 80% vaccination rate target set by Healthy People 2020 [3]. Even considering recent recommendations for only two doses of the HPV vaccine if the first dose is administered before the age of 15, the percentage of females and males aged 13–17 who have received two doses of the vaccine is 52.2% and 39.0%, respectively [4].

A majority of efforts to increase HPV vaccine uptake have rightly focused on improving HPV vaccine uptake among adolescents; given that the immunogenicity of the HPV vaccine is highest when administered during the ages of 11–12 years old and that the vaccine is most effective prior to sexual initiation [5]. However, there is still a tremendous opportunity to improve uptake among young adults who have not been vaccinated against HPV. While the HPV vaccine is recommended until the age of 26 for females and 21 for males (26 for high-risk males), HPV vaccination rates are currently 40.2% and 8.2% for females and males aged 19–26, respectively [6]. In light of this, interventions targeting young adults may also be necessary to increase HPV vaccination rates until we can reach vaccination targets for adolescents [7].

Previous research on predictors of HPV vaccination or vaccination intentions have focused on specific beliefs and attitudes about HPV and the HPV vaccine, or the demographic and social characteristics of HPV vaccinators versus non-vaccinators [8–10]. For example, a recent systematic review highlighted how beliefs that the HPV vaccine is effective or that their child was at risk of contracting HPV are associated with increased HPV vaccine acceptability [9]. The focus of the current research is an exploratory examination of whether there are psychological traits associated with HPV vaccination status and intentions in young adults. In the current study, we focused on psychological traits which are conceptually grouped by an individual’s motivation to: 1) understand health-related information, 2) deliberate, 3) manage uncertainty, and 4) manage threat. People vary in their baseline motivation to meet these needs [11–16], and we hypothesize that variations in these motives may be associated with HPV vaccination status and intentions. If true, these motives would merit consideration when designing interventions to improve HPV vaccine uptake. Below we will describe each of these psychological motivations in more detail.

*Understanding health-related information* refers to the extent to which a person is interested in or able to understand health-related information [12,17]. While almost all major theories of health behavior (e.g., Health Belief Model [18]; Theory of Reasoned Action [19]) presume that having an accurate understanding of risks and benefits is necessary to promote appropriate health behavior, very few studies measure underlying differences in interest in or ability to understand health-related information. Understanding health-related information could include a preference for or motivation to understand scientific, medical, or numeric information. Given that a preponderance of the evidence supports the safety and efficacy of the HPV vaccine [2,20], individuals with greater motivation to understand health-related information may be more likely to either be vaccinated or willing to be vaccinated against HPV.

*Deliberation* is the extent to which a person likes to think about things or engage in effortful cognition (in contrast to relying on their intuition) [21]. Research from the attitudes and persuasion literature, as well as the decision science literature, has highlighted that engaging in more effortful processing frequently leads to better decision-making (i.e., decisions consistent with the evidence or values, e.g., [13,22,23]). However, there is also research highlighting that deliberation sometimes gets in the way of good decision-making and that making decisions based on intuition are sometimes more accurate, particularly when the decision involves complex or large amounts of information [24,25]. Given that information about vaccines can be relatively complex, a person’s propensity to engage in effortful thought or make decisions based on their intuitions could influence HPV vaccination decisions in either direction.

*Managing uncertainty* is the extent to which a person finds ambiguity and uncertainty aversive, and seeks to reduce uncertainty, sometimes at the expense of making a decision that is consistent with the overall evidence [14,26,27]. Uncertainty is an inherent feature in any health-related or medical decision [28]. It is almost impossible to have complete information about a health risk or what can be done to prevent or treat it. For example, a person may be concerned about the uncertainty regarding the likelihood of contracting HPV or about the perceived uncertainty about the efficacy or safety of the HPV vaccine. Consequently, people may feel the need to manage the uncertainty of contracting HPV by being vaccinated, while others may feel uncertain about the efficacy and safety of the HPV vaccine and choose not to be vaccinated.

Finally, *managing threat* is the extent to which a person is sensitive to, and feels the need to deal with, threats [16,29–31]. HPV is inherently a risk and the HPV vaccine is not without risks of adverse events. As a result, a person may feel the need to manage the threat of contracting HPV by being vaccinated, while others may feel the need to manage the threat of experiencing an adverse event and choose to not be vaccinated.

### Hypotheses

Based on the above descriptions of the different motivations, we made the following hypotheses regarding the possible associations between these motivations with HPV vaccination status and intentions (see Table 1 for a summary of psychological traits, scales, and hypotheses for each type of motivation):

**Table 1:** Psychological traits, definitions, scales/measures, and predictions for each type of motivation.

Traits^a^	Definition^b^	Scale/Measure^a^	HPV Prediction^c^
**Motivation: Understand health-related information**			
Scientific curiosity	Interest in science-related information	Single-item Scientific Curiosity measure	Higher understanding of health-related information will be associated with being vaccinated, or being willing to be vaccinated, against HPV (Hypothesis 1).
Scientific intelligence	Understanding of scientific facts and reasoning	Ordinary Science Intelligence 2.0 Scale	
Health literacy	Ability to understand health-related information	Single-item Health Literacy measure	
Numeracy	Interest in and described ability to understand numeric information	Subjective Numeracy Scale-3 (SNS-3) Scale	
**Motivation: Deliberate**			
Cognitive reflection	Ability to override prepotent response	Cognitive Reflection Task 2.0	Higher deliberation (Hypothesis 2a) OR lower deliberation (Hypothesis 2b) could be associated with being vaccinated, or being willing to be vaccinated, against HPV.
Need for cognition	Preference for engaging in effortful cognitive activities	Need for Cognition Scale	
Faith in intuition	Preference for relying on feelings and intuitions for judgments or decisions	Faith in Intuition Scale	
**Motivation: Manage uncertainty**			
Need for cognitive closure	Preference to quickly arrive at and maintain a conclusion	Brief Need for Cognitive Closure Scale	Higher need to manage uncertainty could be associated with either increased (Hypothesis 3a) or decreased (Hypothesis 3b) likelihood of being vaccinated or being willing to be vaccinated against HPV.
Open-minded thinking	Openness to new information and belief updating	Actively Open-Minded Thinking Scale	
**Motivation: Manage threat**			
Emotional reactivity to rare events	Concern about possibility (not probability) of experiencing a rare event	Single-item measure of Emotional Reactivity to Rare Events	Higher need to manage threat could be associated with either increased (Hypothesis 3a) or decreased (Hypothesis 3b) likelihood of being vaccinated or being willing to be vaccinated against HPV.
Social dominance orientation	Preference for clear group boundaries and sensitivity to group-based threats	Social Dominance Orientation Scale	
Belief in a dangerous world	Belief world is generally a dangerous place	Belief in a Dangerous World Scale	

^a^See Measures section for more information.

^b^See Introduction for more information.

^c^See Hypotheses section for more information

#### Understanding health-related information

- **Hypothesis 1:** We hypothesize that individuals with a higher motivation to understand health-related information will likely have a better understanding of the efficacy and safety of the HPV vaccine and threat of HPV infection. As a result, we predict that higher understanding of health-related information will be associated with being vaccinated against HPV or having higher HPV vaccination intentions.

#### Deliberation

- **Hypothesis 2a:** Given the earlier discussion of the literature highlighting that higher deliberation frequently leads to more optimal decision-making (e.g., making a decision consistent with the available scientific evidence), one prediction is that higher deliberation will be associated with being vaccinated against HPV or having higher HPV vaccination intentions.
- **Hypothesis 2b:** Alternatively, given the evidence that under some circumstances (e.g., complex decisions; large amounts of information to consider) people make more optimal decisions using a less deliberative or more intuitive approach, another prediction is that lower deliberation (or more intuitive decision-making) will be associated with being vaccinated against HPV or having higher HPV vaccination intentions.

#### Managing uncertainty and threat

- **Hypothesis 3a:** Contracting HPV is inherently a health risk and there is uncertainty for any person who has sex whether they will contract it. As a result, high motivations to manage uncertainty or threat may be associated with being vaccinated against HPV or having higher HPV vaccination intentions.
- **Hypothesis 3b:** Alternatively, individuals may perceive that there is uncertainty about the efficacy of the HPV vaccine and whether they will experience any adverse effects from the vaccine. If this is the case, then high motivations to manage uncertainty or threat may be associated with not being vaccinated against HPV or having lower HPV vaccination intentions.

In addition to the personality traits linked to these motivations, we also examined whether the associations between these personality traits and HPV vaccine uptake and intentions were moderated by three factors. Gender and race were include as potential moderators given disparities in HPV vaccine uptake based on these characteristics [6]. Insurance status was also included as a potential moderator, since the ability of personality traits to influence HPV vaccine uptake or intentions may only manifest under conditions in which the person has relatively easier access to receiving the HPV vaccine.

## Materials and methods

### Participants

After receiving exempt status from the University of Iowa Biomedical Institutional Review Board, we recruited a stratified random sample of U.S. adults aged 18–26 from Survey Sampling International (SSI). SSI maintains a panel of Internet users recruited through various opt-in methods and uses a probability-weighted random process to identify which panel members should receive different surveys based on sample requirements. To ensure demographic diversity, we established quotas based on both respondent race (thereby approximating the distributions of race in the U.S. population), age (50% 18–21; 50% 22–26), and gender (50% female, 50% male or other). The sampling algorithm continued to route SSI participants to the survey until all quotas were achieved. We recruited over a one-week period in July 2017. Upon completion, participants were entered into both instant-win contests and regular drawings administered by SSI for modest prizes.

### Design and procedure

Eligible participants were given a link to a survey that was programmed using Qualtrics® survey platform. After reading some introductory information about the study, participants answered a screener question indicating whether they were between the ages of 18–26. Participants who were in the eligible age range responded to a variety of questions regarding their HPV vaccination status, intentions to receive the HPV vaccine (if they reported not being vaccinated or did not know), measures of the personality traits linked to each motivation (using previously validated items or scales), and demographics.

### Measures

Study measures and data files are available on the Open Science Framework [32].

#### HPV vaccination status and vaccination intentions

Participants indicated whether they had received the HPV vaccine with “no”, “yes”, and “don’t know/not sure” as response options. Participants who indicated they had been vaccinated were asked follow-up questions regarding how many vaccines in the vaccine series they had received—with 0, 1, 2, 3, and “Don’t know” as response options—and the *main* reason why they were vaccinated, with “my parents had me get the vaccine”, “my doctor recommended it”, “to prevent genital warts”, “to prevent cancer”, “it just seemed like the right thing to do”, and “other” as response options. As a measure of vaccination intentions, participants who indicated that they were not vaccinated or did not know if they were vaccinated were asked how likely they would be to contact their health provider to get the HPV vaccine after they were done with this study. Responses were on a 7-point Likert-type scale with “Not at all likely-1” and “Very likely-7” as the endpoint labels.

#### Understanding health-related information

We measured via four related, but distinct, personality traits for understanding health-related information: scientific curiosity, scientific intelligence, health literacy, and numeracy. Scientific curiosity is the extent to which a person is interested in science-related information and measured via a single item measure, in which participants indicate how interested they are in “scientific research and discoveries” with “1 Not at all interested”; “2 slightly interested”; “3 more than slightly interested but not very interested”; and “4 very interested” as response options [33]. The Ordinary Science Intelligence 2.0 scale was used to measure the extent to which a person understands scientific facts and reasoning [34]. Health literacy is the ability to understand health-related information and was measured via the single-item Health Literacy measure [12]. The 3-item Subjective Numeracy Scale was used to measure the extent to which a person has an interest in and self-described ability to understand numeric information [35].

#### Deliberation

Three personality traits were used to assess motivation to deliberate: cognitive reflection, need for cognition, and faith in intuition. The Cognitive Reflection Task 2.0 was used to measure a person’s ability to override a prepotent response [36]. For example, when asked “If you’re running a race and you pass the person in second place, what place are you in?” people often have the initial intuitive response of ‘first place’, when the correct answer is ‘second place’. The Need for Cognition Scale was used to measure the extent to which a person likes to think about things or engage in effortful cognitive activities [21]. In contrast to cognitive reflection and need for cognition, the Faith in Intuition Scale was used to measure the extent to which a person relies on their feelings and intuitions to make judgments or decisions [37]. It is worth noting that while engaging in cognitive effort and relying on intuition seem conceptually opposed to each other, previous research has demonstrated that they can be orthogonal to each other [37].

#### Managing uncertainty

Two personality traits were used to assess motivation to manage uncertainty: need for cognitive closure and open-minded thinking. The Brief Need for Cognitive Closure Scale was used to measure the extent to which a person tries to reduce uncertainty by quickly arriving at and maintaining a conclusion [38]. In contrast, the Actively Open-Minded Thinking Scale was used to measure the extent to which a person is open to new information and updating their beliefs (i.e., more open to uncertainty) [39].

#### Managing threat

Three personality traits that measured different types of threat sensitivity were used to assess motivation to manage threat: emotional reactivity to rare events, social dominance orientation, and belief in a dangerous world. Emotional reactivity to rare events is the extent to which a person experiences concern about the possibility (not probability) of experiencing extremely rare risks and was measured via a single-item measure [40]. The Social Dominance Orientation Scale was used to measure the extent to which an individual has a preference for groups with clear boundaries and are sensitive to group-based threats [15]. Finally, the Belief in a Dangerous World Scale was used to evaluate the extent to which a person believes the world is generally a dangerous place and is becoming more dangerous [16].

#### Demographics

Participants indicated their gender, age, race/ethnicity, sexual orientation, education, income, and insurance status.

### Data analyses

#### Recoding of measures

Scale responses were averaged to create an aggregate value for subjective numeracy (Cronbach’s α = .92; range: 1–6); need for cognition (Cronbach’s α = .60; range: 1–5); faith in intuition (Cronbach’s α = .84; range: 1–5); need for cognitive closure (Cronbach’s α = .85; range:1.8–6); active open-minded thinking (Cronbach’s α = .84; range: 1.6–9); social dominance orientation (Cronbach’s α = .91; range: 1–10); and belief in a dangerous world (Cronbach’s α = .87; range 1–6). Responses were scored as correct (1) or incorrect (0) and summed for scientific intelligence (range: 0–7) and cognitive reflection (range: 0–4).

#### Statistical analyses

Descriptive analyses were used for the demographic measures and bivariate correlations for the personality traits. One-way ANOVAs were run to test for differences in personality traits based on vaccination status. To test for potential moderation, we tested for interactions between vaccination status and gender (male, female), race (White; non-White), and insurance status (insured, uninsured) on the outcome measures using two-way ANOVAs. A linear regression model was created to determine which psychological traits were predictive of HPV vaccination intentions, with age, gender, race, education, income, and insurance status as covariates. All major analyses were run with and without the 172 respondents who reported that their parents made the decision for them to receive the HPV vaccine. The results were not significantly different between the two sets of analyses, which we will discuss more in the *Discussion* section, so we chose to report the results that include these 172 respondents in order to increase the power of our analyses. All analyses were performed using Stata 14 and all tests of significance were 2-sided and used α = .05.

## Results

### Sample characteristics

Out of 1,674 people who initiated the survey, 1,406 people completed it (an 84% completion rate). Forty-seven responses were excluded due to a reported age outside of the 18–26 year old range and one person was excluded for not responding to the HPV vaccination status item.

Demographics of the remaining 1,358 respondents are shown in Table 2. Demographics were within +/-5% of national rates [41], with the exception of age and education, which can be attributed to the purposive sampling of 18–26 year olds. As can be seen in the “Vaccinated” column, there were similar reported vaccination rates across age and sexual orientation. However, there were noticeable differences across gender, race, education, income, and insurance status. Consistent with national vaccination rates (40.2% and 8.2% for females and males aged 19–26, respectively[6]), a greater percentage of women reported being vaccinated (44.7%) compared to the percentage of men who reported being vaccinated (24.6%). Regarding race and ethnicity, reported vaccination rates were similar across individuals who selected “White”, “African American”, or “Asian/Asian American”, while individuals who selected “Other” as a racial category had lower reported HPV vaccination rates compared to the other three categories. Respondents with less education, earning less than $25,000, and either being uninsured or not knowing their insurance status reported lower vaccination rates relative to respondents in the other groups for those demographics.

**Table 2:** Respondent characteristics.

Characteristic	Total Sample Frequency (%)	Vaccinated Frequency (%)	Unvaccinated Frequency (%)	Unsure Frequency (%)
**Age**				
18–21	686 (50.5%)	247 (36.0%)	177 (25.8%)	262 (38.2%)
22–26	672 (49.5%)	235 (35.0%)	241 (35.9%)	196 (29.2%)
**Gender**				
Male	594 (43.9%)	146 (24.6%)	222 (37.4%)	226 (38.0%)
Female	730 (54.0%)	326 (44.7%)	189 (25.9%)	215 (29.4%)
Transgender/Other	29 (2.1%)	9 (31.0%)	7 (24.1%)	13 (44.8%)
**Ethnicity**				
Hispanic	296 (21.9%)	108 (36.5%)	74 (25.0%)	114 (38.5%)
**Race**^**a**^				
White	1004 (73.9%)	368 (36.7%)	309 (30.8%)	327 (32.6%)
African American	187 (13.8%)	68 (36.4%)	66 (35.3%)	53 (28.3%)
Asian/Asian American	93 (6.8%)	37 (39.8%)	22 (23.7%)	34 (36.6%)
Other	155 (11.4%)	41 (26.5%)	46 (29.7%)	68 (43.9%)
**Sexual orientation**				
Heterosexual	1005 (74.4%)	357 (35.5%)	318 (31.6%)	330 (32.8%)
Homosexual	84 (6.2%)	30 (35.7%)	27 (32.1%)	27 (32.1%)
Bisexual/Other	261 (19.4%)	93 (35.6%)	73 (28.0%)	95 (36.4%)
**Education**				
< Bachelor’s degree	1100 (81.0%)	366 (33.3%)	333 (30.3%)	401 (36.4%)
Bachelor’s degree or higher	258 (19.0%)	116 (45.0%)	85 (33.0%)	57 (22.1%)
**Income**				
<$25,000	428 (31.8%)	132 (30.8%)	128 (29.9%)	168 (39.3%)
$25,000-$74,99	636 (47.2%)	236 (37.1%)	190 (29.9%)	210 (33.0%)
$75,000-$149,000	231 (17.2%)	92 (39.8%)	83 (35.9%)	56 (24.2%)
$150,000 or more	52 (3.9%)	19 (36.5%)	15 (28.9%)	18 (34.6%)
**Insured**				
Yes	1051 (79.1%)	400 (38.1%)	314 (29.9%)	337 (32.1%)
No	195 (14.7%)	52 (26.7%)	74 (38.0%)	69 (35.4%)
Don’t know	83 (6.3%)	19 (22.9%)	21 (25.3%)	43 (51.8%)
**Vaccination decision by parent**				
No		308 (64.7%)		
Yes		172 (35.8%)		
**Number of HPV vaccinations**				
1		85 (17.7%)		
2		116 (24.3%)		
3		206 (43.1%)		
Don’t Know		71 (14.9%)		

NOTE: Reports result only for those respondents who responded to the characteristic. Percentages in the total column represent percent of total sample. Percentages in the “Vaccinated”, “Unvaccinated”, and “Unsure” columns represent percent of respondents for that demographic characteristic (e.g., % of respondents aged 18–21).

^a^Respondents could mark more than one race.

### Understanding health-related information

The small to moderate bivariate correlations among the four measures of understanding health-related information (Table 3) suggests that each of these measures is related to, but distinct from, each other. These correlations make sense given that these measures pertain to interest in or ability to understand different types of information (scientific, medical, numeric). See S1 Table for correlations between all of the personality traits with each other.

**Table 3:** Bivariate correlations among personality measures.

Psychological Motive				
**Understanding**				
	Curiosity	Scientific intelligence	Health literacy	Numeracy
Curiosity	1.00			
Scientific intelligence	.20 (< .001)	1.00		
Health literacy	.19 (< .001)	.21 (< .001)	1.00	
Subjective numeracy	.32 (< .001)	.30 (< .001)	.35 (< .001)	1.00
**Deliberation**				
	Cognitive reflection	Need for Cognition	Faith in intuition	
Cognitive reflection	1.00			
Need for cognition	-.22 (< .001)	1.00		
Faith in intuition	-.05 (.058)	-.01 (.692)	1.00	
**Managing Uncertainty**				
	Cognitive closure	Open mindedness		
Cognitive closure	1.00			
Open mindedness	-.14 (< .001)	1.00		
**Managing Threat**				
	Emotional reactivity to rare events	Social dominance orientation	Belief in a dangerous world	
Emotional reactivity to rare events	1.00			
Social dominance orientation	.09 (.002)	1.00		
Belief in a dangerous world	.16 (< .001)	-.00 (.874)	1.00	

*Note*: Cells in grey indicate p-value ≤ .05.

There was a statistically significant association of vaccination status with all four understanding of health-related information personality traits (Table 4). Specifically, *vaccinators* (respondents who reported being vaccinated) had higher scientific curiosity, scientific intelligence, health literacy, and subjective numeracy relative to *non-vaccinators* (respondents who did not report being vaccinated) or *the unsure* (respondents who were unsure if they were vaccinated or not). Our linear regression model, which controlled for relevant demographic characteristics, revealed that scientific curiosity and subjective numeracy, but not scientific intelligence or health literacy, were significantly associated with increased intentions to contact their health care provider to receive the HPV vaccine after the conclusion of the study (Table 5).

**Table 4:** One-way ANOVAs testing for differences in measures based on vaccination status.

Psychological Motives	Vaccinated Mean (SD)	Unvaccinated Mean (SD)	Unsure Mean (SD)	F-value (p-value)	Effect size ƞ^2^ (95% CI)
**Understanding**					
Scientific Curiosity	3.07 (0.88)	2.92 (0.99)	2.95 (0.96)	3.50 (.030)	.005 (.000, .015)
Scientific Intelligence	4.44 (1.88)	4.04 (1.94)	3.98 (1.93)	7.83 (< .001)	.011 (.002, 0.24)
Health Literacy	4.04 (1.04)	3.85 (1.22)	3.67 (1.12)	13.07 (< .001)	.019 (.007, .035)
Subjective Numeracy	4.17 (1.33)	4.03 (1.34)	3.85 (1.27)	6.82 (.001)	.010 (.002, .022)
**Deliberation**					
Cognitive Reflection	1.79 (1.23)	1.63 (1.27)	1.88 (1.22)	4.65 (.010)	.007 (.000, .017)
Need for Cognition	2.56 (0.77)	2.64 (0.73)	2.66 (0.65)	2.27 (.104)	.003 (.000, .011)
Faith in Intuition	3.64 (0.84)	3.54 (0.89)	3.48 (0.79)	4.21 (.015)	.006 (.000, .016)
**Managing Uncertainty**					
Need for Cognitive Closure	3.74 (0.64)	3.70 (0.64)	2.66 (0.65)	2.77 (.063)	.004 (.000, .013)
Open-Minded Thinking	5.83 (1.19)	5.75 (1.27)	5.80 (1.19)	0.46 (.633)	.001 (.000, .005)
**Managing Threat**					
Emotional Reactivity to Rare Events	2.73 (1.28)	2.84 (1.29)	2.67 (1.22)	2.17 (.115)	.003 (.000, .011)
Social Dominance Orientation	3.57 (2.02)	3.69 (1.92)	3.61 (1.86)	0.49 (.611)	.001(.000, .005)
Belief in a Dangerous World	3.67 (0.80)	3.65 (0.79)	3.63 (0.70)	0.33 (.722)	.000 (.000, .004)

*Note*: Shaded areas indicate where there is a statistically significant difference between groups.

**Table 5:** Linear regression results for HPV vaccination intentions (N = 1041).

	Coef. (95% CI)	β	p-value
**Understanding**			
Scientific Curiosity	**0.23 (0.10, 0.35)**	**0.11**	**< .001**
Scientific Literacy	-0.04 (-0.11, 0.03)	-0.04	.309
Health Literacy	0.06 (-0.05, 0.17)	0.03	.312
Subjective Numeracy	**0.17 (0.06, 0.27)**	**0.11**	**.002**
**Deliberation**			
Cognitive Reflection	**-0.17 (-0.27, -0.07)**	**-0.11**	**.001**
Need for Cognition	0.13 (-0.05, 0.31)	0.05	.154
Faith in Intuition	0.08 (-0.08, 0.23)	0.03	.333
**Managing Uncertainty**			
Need for Cognitive Closure	**0.21 (0.00, 0.42)**	**0.06**	**.046**
Open-Minded Thinking	**-0.31 (-0.43, -0.19)**	**-0.19**	**< .001**
**Managing Threat**			
Emotional Reactivity to Rare Events	**0.20 (0.10, 0.29)**	**0.13**	**< .001**
Social Dominance Orientation	-0.01 (-0.08, 0.06)	-0.01	.796
Belief in a Dangerous World	-0.14 (-0.30, 0.01)	-0.06	.075
**Demographics**			
Age	0.01 (-0.04, 0.06)	0.01	.709
Gender	-0.18 (-0.42, 0.07)	-0.05	.156
Race	**-0.33 (-0.59, -0.07)**	**-0.07**	**.013**
Education	0.05 (-0.02, 0.13)	0.05	.156
Income	-0.05 (-0.11, 0.01)	-0.05	.107
Insurance status	0.24 (-0.04, 0.51)	0.05	.096
Constant	**2.74 (1.01, 4.46)**		**.002**

*Note*: Gender (0 = male, 1 = female), race (0 = White, 1 = non-White), and insurance status (0 = no or don’t know, 1 = yes). Age, education, and income analyzed as continuous variables. Values in bold indicate p-value ≤ .05. Unlike the vaccination status results, results did not differ based on gender, so we present results collapsed across gender.

While there was only a statistically significant interaction between vaccination status and gender for scientific intelligence (Table 6), there were interesting differences between men and women across the understanding measures. While female vaccinators had higher means scores on all four understanding measures compared to female non-vaccinators and the unsure, this pattern was only significant for males for health literacy and subjective numeracy (Table 7). There was no evidence of moderation based on race (all interaction term *p*s>.52) or insurance status (*p*s>.22).

**Table 6:** Two-way ANOVAs testing associations and interactions of vaccination status and gender.

Psychological Motives	Vaccination Status F-statistic (p-value)	Gender F-statistic (p-value)	Interaction F-statistic (p-value)
**Understanding**			
Scientific Curiosity	**3.76 (.023)**	**6.26 (.013)**	1.23 (.291)
Scientific Intelligence	2.07 (.123)	**33.15 (< .001)**	**5.22 (.006)**
Health Literacy	**9.26 (< .001)**	2.64 (.104)	0.33 (.717)
Subjective Numeracy	**11.44 (< .001**)	**43.68 (< .001)**	0.04 (.958)
**Deliberation**			
Cognitive Reflection	**4.89 (.008)**	**5.70 (.017)**	**4.15 (.016)**
Need for Cognition	0.26 (.770)	**8.20 (.004)**	**5.52 (.004)**
Faith in Intuition	**3.19 (.041)**	**5.07 (.025)**	2.54 (.079)
**Managing Uncertainty**			
Need for Cognitive Closure	**5.15 (.006)**	0.16 (.691)	**5.41 (.005)**
Open-Minded Thinking	0.29 (.746)	**4.09 (.043)**	**12.49 (< .001)**
**Managing Threat**			
Emotional Reactivity to Rare Events	2.25 (.106)	0.43 (.514)	**11.64 (< .001)**
Social Dominance Orientation	2.05 (.129)	**85.53 (< .001)**	**7.96 (< .001)**
Belief in a Dangerous World	0.29 (.752)	**34.23 (< .001)**	0.00 (.996)

*Note*: Values in bold indicate p-value ≤ .05.

**Table 7:** Psychological trait differences based on vaccination status and gender.

	Men			Women		
Psychological Motives	Vaccinated n = 146 Mean (SD)	Unvaccinated n = 222 Mean (SD)	Unsure n = 226 Mean (SD)	Vaccinated n = 326 Mean (SD)	Unvaccinated n = 189 Mean (SD)	Unsure n = 215 Mean (SD)
**Understanding**						
Scientific Curiosity	3.10 (0.87)	2.97 (0.98)	3.05 (0.92)	3.06 (0.89)	2.84 (1.01)	2.82 (1.01)
Scientific Intelligence	3.68 (1.94)	3.87 (1.97)	3.78 (1.99)	4.78 (1.77)	4.22 (1.91)	4.17 (1.77)
Health Literacy	3.94 (1.11)	3.85 (1.21)	3.64 (1.16)	4.10 (0.98)	3.88 (1.12)	3.74 (1.04)
Subjective Numeracy	4.51 (1.31)	4.26 (1.39)	4.11 (1.30)	4.04 (1.30)	3.79 (1.23)	3.59 (1.20)
**Deliberation**						
Cognitive Reflection	1.48 (1.38)	1.60 (1.28)	1.91 (1.25)	1.92 (1.14)	1.68 (1.26)	1.89 (1.17)
Need for Cognition	2.77 (0.71)	2.68 (0.79)	2.64 (0.79)	2.48 (0.76)	2.60 (0.66)	2.67 (0.67)
Faith in Intuition	3.67 (0.80)	3.45 (0.89)	3.44 (0.78)	3.63 (0.84)	3.66 (0.89)	3.58 (0.75)
**Managing Uncertainty**						
Need for Cognitive Closure	3.85 (0.72)	3.65 (0.66)	3.61 (0.54)	3.70 (0.58)	3.77 (0.60)	3.68 (0.54)
Open-Minded Thinking	5.39 (1.08)	5.79 (1.26)	5.82 (1.18)	6.01 (1.18)	5.69 (1.29)	5.71 (1.14)
**Managing Threat**						
Emotional Reactivity to						
Rare Events	3.07 (1.36)	2.85 (1.31)	2.52 (1.16)	2.58 (1.22)	2.86 (1.26)	2.85 (1.26)
Social Dominance Orientation	4.66 (1.79)	3.99 (1.91)	3.99 (1.85)	3.09 (1.92)	3.36 (1.88)	3.27 (1.76)
Belief in a Dangerous World	3.50 (0.70)	3.54 (0.75)	3.52 (0.72)	3.75 (0.83)	3.79 (0.82)	3.76 (0.63)

*Note*: Shaded areas indicate where there is a statistically significant difference between groups.

### Deliberation

Cognitive reflection and need for cognition shared a small, negative correlation (Table 3). Consistent with previous research [37], faith in intuition was uncorrelated with need for cognition and cognitive reflection (Table 3).

There were significant associations of vaccination status with cognitive reflection and faith in intuition. Specifically, vaccinators had the highest faith in intuition mean, while the unsure group had the highest cognitive reflection mean (Table 4). Cognitive reflection, but not need for cognition or faith in intuition, was significantly associated with decreased vaccination intentions (Table 5).

There were statistically significant interactions between vaccination status and gender for cognitive reflection and need for cognition, with a marginally significant interaction for faith in intuition (Table 6). Specifically, male vaccinators had lower cognitive reflection and higher faith in intuition compared to male non-vaccinators or the unsure. Female vaccinators had lower need for cognition compared to female non-vaccinators or the unsure (see Table 7). With one exception (vaccination status and education interaction for need for cognition, p = .011), there were no significant interactions between vaccination status with race or insurance status (*p*s>.07).

### Managing uncertainty

Need for cognitive closure and open-minded thinking shared a small, negative correlation (Table 3). There were no statistically significant associations of vaccination status with need for cognitive closure or open-minded thinking (Table 4), but both measures were associated with vaccination intentions, with higher need for cognitive closure and lower open-minded thinking being associated with increased vaccination intentions (Table 5).

Vaccination status did interact with gender for both need for cognitive closure and open-minded thinking (Table 6). Male vaccinators had higher need for cognitive closure compared to the other two groups, while there was no association between vaccination status and need for cognitive closure for females (Table 7). Interestingly, female vaccinators had higher open-minded thinking compared to female non-vaccinators or the unsure, while this pattern was reversed for males (Table 7). Race and insurance status did not interact with vaccination status for need for cognitive closure or open-minded thinking (*p*s>.36).

### Managing threats

Emotional reactivity to rare events had small, positive correlations with social dominance orientation and belief in a dangerous world, but the latter two were not significantly correlated (Table 3). There were no statistically significant associations of vaccination status with emotional reactivity to rare events, social dominance orientation, or belief in a dangerous world (Table 4) and only emotional reactivity to rare events was significantly associated with vaccination intentions (Table 5).

There were statistically significant interactions between vaccination status and gender for emotional reactivity to rare events and social dominance orientation, but not belief in a dangerous world (Table 6). Specifically, while male vaccinators had greater mean scores for emotional reactivity to rare events and social dominance orientation compared to male non-vaccinators and the unsure, while this pattern was reversed for women (Table 7). With one exception—a non-interesting interaction between vaccination status and race for emotional reactivity (*p* = .006), where vaccinator means fell between non-vaccinators and the unsure—there were no statistically significant interactions between vaccination status with race or insurance status (*p*s>.16).

## Discussion

The goal of the current study was to provide an exploratory examination of whether certain psychological traits may be associated with HPV vaccination status and intentions. Overall, the results suggest that there are important differences in psychological traits between individuals who report being vaccinated against HPV and those who do not report being vaccinated, but more interestingly, there were different motivational profiles between men and women. Women who reported being vaccinated had higher interest in and ability to understand health-related information across all four measures, higher open-minded thinking, and lower need for cognition and emotional reactivity to rare events. In contrast, men who reported being vaccinated had higher motivation to understand some health-related information (health and numeric, but not scientific), but were primarily motivated to reduce uncertainty (higher need for cognitive closure and lower open-minded thinking), threats (group-based threats (i.e., social dominance orientation) and rare events), and deliberation (lower cognitive reflection and higher faith in intuition). These results suggest that while women who receive the HPV vaccine may do so based on informational evidence, men who receive the HPV vaccine may be more motivated based on responsiveness to factors that shape their affective or intuitive responses. We hypothesize that one reason for these differences may be that young women have frequent interactions with the health care system (relative to young men) and are more engaged in their health care [42,43], whereas men usually wait until there is a perceived problem before seeking medical care [44,45].

Regarding vaccination intentions, we found that scientific curiosity, subjective numeracy, need for certainty (greater need for cognitive closure and lower open-minded thinking), being less cognitively reflective, and being sensitive to rare events were predictive of intentions to contact their health care provider to get vaccinated after the study ended. These results suggest that people who are most open to getting vaccinated against HPV have an interest in scientific and numeric information, but are motivated to respond to threats in a way that reduces uncertainty. This has implications for health communication around HPV vaccination, suggesting that information should be communicated in a way that highlights the risks associated with HPV and reduces uncertainty about the HPV vaccine (e.g., highlighting that the HPV vaccine has received more testing to verify safety and efficacy than any other vaccine).

One decision we made for this study was to include respondents whose parents made a decision for them to get vaccinated. As noted, the results of the analyses did not significantly differ whether they were included or not. One reason why the exclusion of these individuals may not have dramatically changed the results is that personality traits have relatively high heritability coefficients [46]. In other words, the respondents and their parents likely have high overlaps in personality—due to both genetics and shared environments—and that the same psychological traits in motivation that promote HPV vaccine uptake for young adults may be the same psychological traits that promote HPV vaccine decisions among parents. While this explanation is post-hoc, our results provide suggestive evidence for replicating the current study with parents to see if similar results are obtained.

The primary strength of the current study is that it provides a novel demonstration of how HPV vaccine status and intentions are associated with variations in psychological traits using a large, demographically diverse national sample of young adults. However, this study has its limitations. One such limitation is the use of an online sample. While not entirely representative of the U.S. population since it excludes individuals without access to the internet, we were able to recruit a relatively large, demographically-diverse sample with sizeable portions of the sample being people who might typically be excluded from online studies, including people with lower incomes (31.8% of sample made $25,000 or less) and less education (34.9% of our sample had a high school education or less). Another limitation was our reliance on self-reported vaccination status. That said, we were primarily interested in *perceived* vaccination status, since perceived need for vaccination is more likely to drive vaccination intentions rather than the actual need for vaccination. A final limitation is the use of a cross-sectional design, limiting inferences of causality. While a prospective, longitudinal study would be ideal for measuring the associations between psychological traits and HPV vaccine uptake, one would have to measure the psychological traits at either a young age prior to being eligible to receive the HPV vaccine (i.e., 9 or 10 years old, or earlier if at high risk of contracting HPV), or when the individual is able to legally make their own healthcare decisions (i.e., age 18), and follow the individual until they were no longer eligible to receive the HPV vaccine (i.e., 12–15 years). Both of these designs would prove challenging unless part of a larger, long-term study.

The limitations of this study indicate the need for continued research on the psychological traits associated with HPV vaccine uptake. While this study highlights the importance of considering psychological traits, a follow-up study could provide a more rigorous test by utilizing medical records to identify young adults who have received the HPV vaccine and those who have not and sending surveys that includes the measures from the current study. As previously mentioned, another follow-up study could be conducting a modified version of the current study with parents to examine the extent to which the current results extend to surrogate decision-making for HPV vaccination. Finally, other research could examine the generalizability of the current findings to other vaccines. HPV vaccines represent an interesting class of vaccine, preventing both a sexually transmitted infection and cancers that can occur as a result of infection. Both the infection and cancer are emotion-laden outcomes, which could make the psychological traits associated with vaccination with the HPV vaccine different from other vaccines. Alternatively, these differences in psychological traits may be associated with the uptake of vaccines more broadly.

## Conclusion

The current study provides some preliminary evidence that psychological differences in motivations to understand health-related information, deliberate, and manage uncertainty and threat are associated with HPV vaccination status and intentions. Our results suggest that improving HPV vaccine uptake should focus on providing information in a way that emphasizes the harms associated with HPV and reduces uncertainty surrounding the efficacy and safety of the HPV vaccine.

## Supporting information

S1 Table Bivariate correlations among all personality trait measures. *Note*: Cells in grey indicate p-value ≤ .05.
(DOCX)
