# Supplementary material for: Cross-sectional associations between psychological traits, and HPV vaccine uptake and intentions in young adults from the United States
Source: PLoS One. 2018 Feb 23;13(2):e0193363. doi: 10.1371/journal.pone.0193363 (PMC5825097; doi:10.1371/journal.pone.0193363)
Supplement: S1 Table — Note: Cells in grey indicate p-value ≤ .05. (DOCX) [file pone.0193363.s001.docx]

**S1 Table. Bivariate correlations among all personality trait measures.**

|  | Curiosity | Scientific intelligence | Health literacy | Numeracy | Cognitive reflection | Need for cognition | Intuition | Cognitive closure | Open mindedness | Emotional reactivity | Social dominance orientation | Belief in dangerous world |
| --- | --- | --- | --- | --- | --- | --- | --- | --- | --- | --- | --- | --- |
| Curiosity | - |  |  |  |  |  |  |  |  |  |  |  |
| Scientific intelligence | .20 (<.001) | - |  |  |  |  |  |  |  |  |  |  |
| Health literacy | .19  (<.001) | .21  (<.001) | - |  |  |  |  |  |  |  |  |  |
| Numeracy | .32  (<.001) | .30  (<.001) | .35  (<.001) | - |  |  |  |  |  |  |  |  |
| Cognitive reflection | .08  (.003) | .34  (<.001) | .03  (.327) | .17  (<.001) | - |  |  |  |  |  |  |  |
| Need for cognition | -.26  (<.001) | -.35  (<.001) | -.18  (<.001) | -.33  (<.001) | -.22  (<.001) | - |  |  |  |  |  |  |
| Intuition | .08  (.003) | .05  (.079) | .24  (<.001) | .16  (<.001) | -.05  (.058) | -.01  (.692) | - |  |  |  |  |  |
| Cognitive closure | .05  (.048) | .01  (.715) | .16  (<.001) | .15  (<.001) | -.10  (<.001) | .18  (<.001) | .36  (<.001) | - |  |  |  |  |
| Open mindedness | .23  (<.001) | .43  (<.001) | .16  (<.001) | .22  (<.001) | .25  (<.001) | -.38  (<.001) | -.09  (<.001) | -.14  (<.001) | - |  |  |  |
| Emotional reactivity | -.00  (.930) | -.15  (<.001) | .09  (.001) | .02  (.486) | -.19  (<.001) | .16  (<.001) | .16  (<.001) | .18  (<.001) | -.22  (<.001) | - |  |  |
| Social dominance orientation | -.16  (<.001) | -.34  (<.001) | -.16  (<.001) | -.14  (<.001) | -.19  (<.001) | .28  (<.001) | -.11  (<.001) | .03  (.346) | -.46  (<.001) | .09  (.002) | - |  |
| Belief in dangerous world | -.07  (.014) | -.10  (<.001) | .03  (.347) | -.16  (<.001) | -.06  (.042) | .14  (<.001) | .21  (<.001) | .14  (<.001) | -.20  (<.001) | .16  (<.001) | -.00  (.874) | - |

*Note:* Cells in grey indicate p-value ≤ .05.
